# The Distribution of Prion Protein Allotypes Differs Between Sporadic and Iatrogenic Creutzfeldt-Jakob Disease Patients

**DOI:** 10.1371/journal.ppat.1005416

**Published:** 2016-02-03

**Authors:** Roger A. Moore, Mark W. Head, James W. Ironside, Diane L. Ritchie, Gianluigi Zanusso, Young Pyo Choi, Suzette A. Priola

**Affiliations:** 1 Rocky Mountain Laboratories, National Institute of Allergy & Infectious Diseases, National Institutes of Health, Hamilton, Montana, United States of America; 2 National CJD Research & Surveillance Unit, Centre for Clinical Brain Sciences, School of Clinical Sciences, University of Edinburgh, Edinburgh, United Kingdom; 3 Department of Neurological and Movement Sciences, University of Verona, Verona, Italy; 4 Department of Neural Development and Disease, Korea Brain Research Institute, Daegu, Republic of Korea; Creighton University, UNITED STATES

## Abstract

Sporadic Creutzfeldt-Jakob disease (sCJD) is the most prevalent of the human prion diseases, which are fatal and transmissible neurodegenerative diseases caused by the infectious prion protein (PrP^Sc^). The origin of sCJD is unknown, although the initiating event is thought to be the stochastic misfolding of endogenous prion protein (PrP^C^) into infectious PrP^Sc^. By contrast, human growth hormone-associated cases of iatrogenic CJD (iCJD) in the United Kingdom (UK) are associated with exposure to an exogenous source of PrP^Sc^. In both forms of CJD, heterozygosity at residue 129 for methionine (M) or valine (V) in the prion protein gene may affect disease phenotype, onset and progression. However, the relative contribution of each PrP^C^ allotype to PrP^Sc^ in heterozygous cases of CJD is unknown. Using mass spectrometry, we determined that the relative abundance of PrP^Sc^ with M or V at residue 129 in brain specimens from MV cases of sCJD was highly variable. This result is consistent with PrP^C^ containing an M or V at residue 129 having a similar propensity to misfold into PrP^Sc^ thus causing sCJD. By contrast, PrP^Sc^ with V at residue 129 predominated in the majority of the UK human growth hormone associated iCJD cases, consistent with exposure to infectious PrP^Sc^ containing V at residue 129. In both types of CJD, the PrP^Sc^ allotype ratio had no correlation with CJD type, age at clinical onset, or disease duration. Therefore, factors other than PrP^Sc^ allotype abundance must influence the clinical progression and phenotype of heterozygous cases of CJD.

## Author Summary

In Creutzfeldt-Jakob disease (CJD), heterozygosity at residue 129 for methionine or valine in normal prion protein may affect disease phenotype, onset and progression. However, the relative contribution of each prion protein allotype to the infectious, disease associated form of prion protein (PrP^Sc^) is unknown. Here we report the novel observation that in heterozygous cases of sporadic CJD the PrP^Sc^ allotype ratio is highly variable. This case-by-case variability is consistent with the origin of sporadic CJD being the spontaneous, but random, misfolding of either host prion protein allotype into infectious PrP^Sc^. By contrast, in heterozygous cases of iatrogenic CJD in the United Kingdom resulting from exposure to contaminated human growth hormone, the PrP^Sc^ allotype ratio is much more homogeneous and consistent with exposure to infectious PrP^Sc^ containing valine at residue 129. Surprisingly, the PrP^Sc^ allotype ratio did not correlate with disease onset or duration in either disease type. Thus, factors other than PrP^Sc^ allotype ratio likely influence the clinical progression of heterozygous cases of CJD. Moreover, our results suggest that the ratio of methionine to valine in PrP^Sc^ may be a means of determining the origin of prion infection.

## Introduction

Prion diseases are fatal neurodegenerative disorders affecting humans and various other mammals. They are associated with the misfolding of monomeric prion protein (PrP^C^) into a pathological isoform termed PrP^Sc^ that is partially protease resistant, aggregated, and infectious. Human prion diseases include Creutzfeldt-Jakob disease (CJD), Gerstmann-Straussler-Scheinker syndrome (GSS), kuru, fatal familial insomnia (FFI) (for review, see [1]), PrP cerebral amyloid angiopathy [2] and variably protease-sensitive prionopathy [1, 3]. Although CJD occurs in sporadic, genetic, and iatrogenic forms, the most common form is sporadic CJD (sCJD), which occurs at approximately 1–2 cases per million people per year in any given population. Acquired forms of CJD such as kuru, variant CJD, and iatrogenic CJD (iCJD), represent a smaller percentage of all CJD cases.

CJD is associated with a wide diversity of clinicopathological features [1, 4, 5], but the factors that determine these different CJD phenotypes are still being elucidated. Sporadic CJD is classified by a neuropathological profile that appears to correlate with the biochemical properties of PrP^Sc^ [4–7] as well as the sequence of the patient prion protein gene (*PRNP)* at codon 129. All three genotypes of a naturally occurring amino acid polymorphism at *PRNP* codon 129 are found in CJD: homozygous methionine (MM) or valine (VV), and heterozygous (MV). Biochemically, the two major PrP^Sc^ types associated with CJD, termed Type 1 and Type 2, can be distinguished by the molecular mass of PrP^Sc^ following protease digestion. Type 1 PrP^Sc^ has a protease-resistant molecular mass of approximately 21 kDa while a molecular mass of approximately 19 kDa is characteristic of Type 2 PrP^Sc^ [1]. Thus, sCJD can occur with six genotype/PrP^Sc^ type combinations: MM1, MM2, MV1, MV2, VV1, and VV2. These correspond well to the differing clinicopathological presentation found in patients in the six well-recognized sCJD phenotypic subtypes: MM1/MV1, MM2 cortical, MM2 thalamic, MV2, VV1 and VV2 [1, 8, 9]. Based on these criteria, as well as transmission studies in non-human primates [10] and transgenic mice expressing different human *PRNP* genotypes [11, 12], 5 major strains of sCJD have been proposed: MM1/MV1, MV2/VV2, MM2c, MM2t and VV1 [5]. PrP^Sc^ type and *PRNP* genotype are therefore believed to be two major factors influencing CJD phenotype and pathogenesis.

Although not causing disease itself, the presence of M or V at residue 129 in PrP^C^ does affect many aspects of prion disease susceptibility and phenotype [1, 4–9, 13]. For example, in a familial form of human prion disease associated with an aspartic acid to asparagine mutation at residue 178 (D178N), a methionine in cis at position 129 manifests as FFI, while a valine in cis at position 129 manifests as CJD [14]. The codon 129 genotype also affects susceptibility to CJD. Methionine and valine homozygosity at codon 129 are overrepresented in sCJD patients when compared to the general population, whereas MV heterozygosity at codon 129 is underrepresented [15–18]. A similar allelic distribution has been observed in human growth hormone associated cases of iCJD in France [19]. The predisposition towards homozygosity in CJD is even more pronounced in variant CJD, in which all probable and definite clinical cases reported to date have been MM homozygous [16]. By contrast, MM homozygosity is less common in human growth hormone associated cases of iCJD in the United Kingdom (UK) in which heterozygosity and VV homozygosity appear to predominate [19, 20]. Thus, the epidemiological data suggest that the effect of homozygosity or heterozygosity at codon 129 on CJD pathogenesis varies depending upon the type of CJD.

There can be considerable variation within heterozygous MV1 and MV2 sCJD patients with regard to disease duration and onset [6, 9]. This variability may be due to multiple factors including the relative abundance of different populations of PrP^Sc^. For example, in the brain the amount of protease-sensitive PrP^Sc^, a conformationally distinct population of PrP^Sc^ that is aggregated but susceptible to digestion with proteinase K [21], has been correlated with disease progression rate. Multiple forms of PrP^Sc^, including not only Type 1 and Type 2 but also PrP^Sc^ with atypical N-termini, are often found within the same sCJD brain [22, 23] suggesting that PrP^Sc^ type variation may be involved. PrP^Sc^ with either M or V at residue 129 (PrP^Sc^-M129 and PrP^Sc^-V129, respectively) has also been identified in brain material from two different heterozygous sCJD patients [24, 25]. This latter observation is particularly intriguing since the presence of two different PrP allotypes in the same brain can often lead, in a dose-dependent manner, to inefficient PrP^Sc^ formation and increased disease incubation times [26, 27]. Thus, a ready explanation for the variability in disease onset and duration in heterozygous cases of CJD would be quantitative differences between PrP^Sc^ allotypes containing M or V at residue 129. However, the relative abundance of each PrP^Sc^ allotype in MV heterozygous cases of sCJD is unknown.

In order to determine whether differences in CJD phenotype correlated with differences in the relative amounts of PrP^Sc^-M129 and PrP^Sc^-V129, we extracted protease-resistant PrP^Sc^ from brain tissue of multiple sCJD and iCJD patients heterozygous for M and V at codon 129 in *PRNP*. Tandem mass spectrometry was then used to differentiate peptides containing M at residue 129 from those containing V. Our results show that the relative abundance of PrP^Sc^-M129 and PrP^Sc^-V129 was highly variable between individual sCJD cases. Furthermore, the PrP^Sc^ allotype ratio differed between the sCJD and iCJD patient groups and did not correlate with CJD type, age at clinical onset, or disease duration.

## Results

### Differentiation of M and V at residue 129 in human PrP peptides by MS

**Fig 1:**
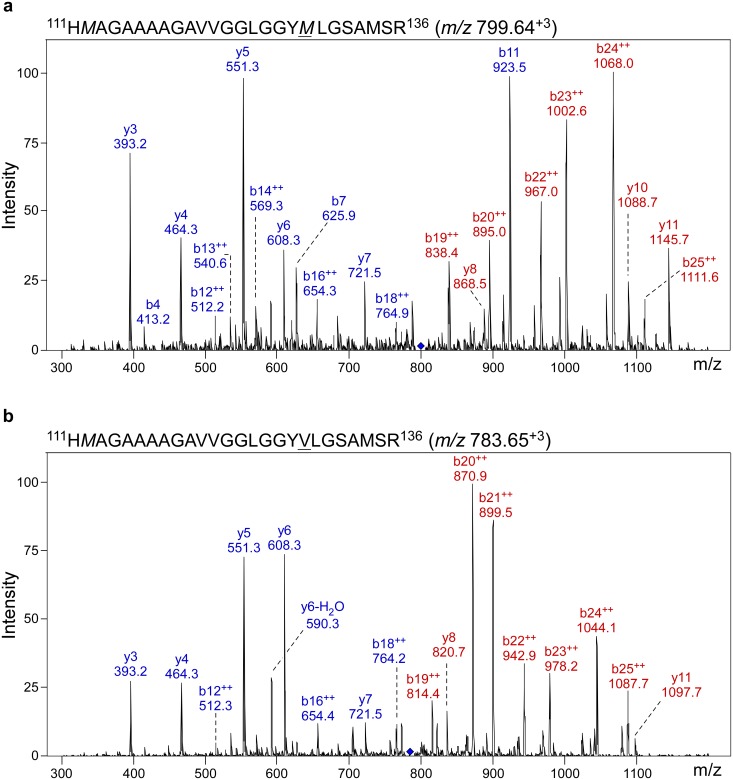
Methionine and valine polymorphism at residue 129 in human PrP can be distinguished by mass spectrometry. Representative MS/MS spectra of human PrP peptide 111–136 with methionine (a) or valine (b) at residue 129 (underlined). The spectra are derived from a PK-treated, PTA-precipitated brain homogenate from sCJD case 10. The PrP111-136 MS/MS spectra were extracted from the raw data using the Data Analysis program v4.0 provided by Agilent. Ion scores of 130 and 127 were assigned by MASCOT for the M129 and V129 PrP111-136 peptides, respectively. Peaks were visualized using Scaffold v4.3 (Proteome Software, Portland, OR). The peptide sequence, charge state and observed *m/z* value for the MS1 ion are shown above each mass spectrum. Italicized methionine residues 112 and 129 in panel A and methionine 112 in panel B are oxidized in this representative example. Those fragment ions for which the *m/z* value changed depending upon the polymorphism at residue 129 are shown in red and those ions with *m/z* values unaffected by the changes at residue 129 are shown in blue. The *m/z* values shown are the experimentally observed values and deviate slightly from the values calculated *in silico* according to the mass accuracy of the instrument used in this study.

In previous MS-based analysis of purified hamster and mouse PrP^Sc^, we found that one of the most commonly identified PrP peptides spanned residues 111–136 (PrP111-136) [28, 29]. This peptide has also been identified by MS in PrP^Sc^ purified from sheep scrapie [30]. In order to determine if MS analysis could also detect PrP111-136 in human PrP^Sc^, PK treatment followed by PTA precipitation was used to isolate PrP^Sc^ from a sample of brain homogenate from a heterozygous case of sCJD. The same procedure was used on brain samples from several non-CJD neurological controls and the samples were compared. Only 5 PrP-specific peptides were found in one of the 3 non-CJD controls, indicating that the PrP^Sc^ enrichment protocol used yielded little or no PrP^C^. By contrast, 525 total PrP peptides were found in the sCJD sample the most common of which are shown in S1 Table. Many of these peptides spanned the region of PrP from residues 111–136 and showed variable levels of methionine oxidation (S1 Table). Importantly, the MV polymorphism at residue 129 was distinguishable based upon the unique fragmentation patterns of peptides containing either M or V (Fig 1a and 1b). The same PrP111-136 peptides were also found, albeit with a much lower rate of methionine sulfoxidation, using rHuPrP-M129 and rHuPrP-V129 purified from *E*. *coli* (S1a and S1b Fig). Analysis of synthetic PrP111-136 peptides containing either M or V at residue 129 confirmed that each peptide yielded distinctive spectra with precise differences between MS/MS fragments corresponding to the presence of either M or V at residue 129 (S1c and S1d Fig). Finally, MS analysis using purified rHuPrP demonstrated that PrP111-136 peptides containing M or V at residue 129 could be differentiated with 100% specificity (S2 Fig).

### Semi-quantitative analysis of peptides containing M or V at residue 129 in human PrP

The detection of peptides with either M or V at residue 129 in PrP^Sc^ from a heterozygous case of sCJD (Fig 1) confirmed earlier studies demonstrating the presence of both PrP^Sc^ allotypes in two cases of heterozygous sCJD [24, 25]. Therefore, both PrP^Sc^-M129 and PrP^Sc^-V129 might contribute to sCJD pathogenesis. However, the relative abundance and thus potential extent of the contribution of each allotype to disease pathogenesis is unknown. Spectral counting, which is defined as the number of MS spectra identified for a protein, is a semi-quantitative technique that is often used as a practical method for estimating protein abundance [31]. It provides a robust label-free estimate for comparing the relative abundance of proteins in or between sample groups [32]. We first determined the quantitative relationship between PrP concentration and spectral counts using MS analysis of a solution of purified rHuPrP stoichiometrically adjusted to contain different molar ratios of rHuPrP-M129 and V129 (S2 Fig). There was a good correlation between spectral counts and the concentration of rHuPrP-M129 (R^2^ = 0.89). At a molar ratio of 50:50, 56 ± 2% of the PrP111-136 peptides were identified as M129 while approximately 44 ± 2% contained V129. Thus, spectral counting allowed us to estimate the relative abundance of M or V at residue 129 in a heterozygous mixture of human PrP, albeit with a slight bias towards detection of the M versus the V PrP111-136 peptide. Therefore, we used a spectral counting approach to semi-quantitatively determine the relative amounts of PrP^Sc^-M129 and PrP^Sc^-V129 in brain tissue derived from heterozygous cases of sCJD.

### Variable abundance of M and V at residue 129 in PrP^Sc^ isolated from sCJD brain

**Fig 2:**
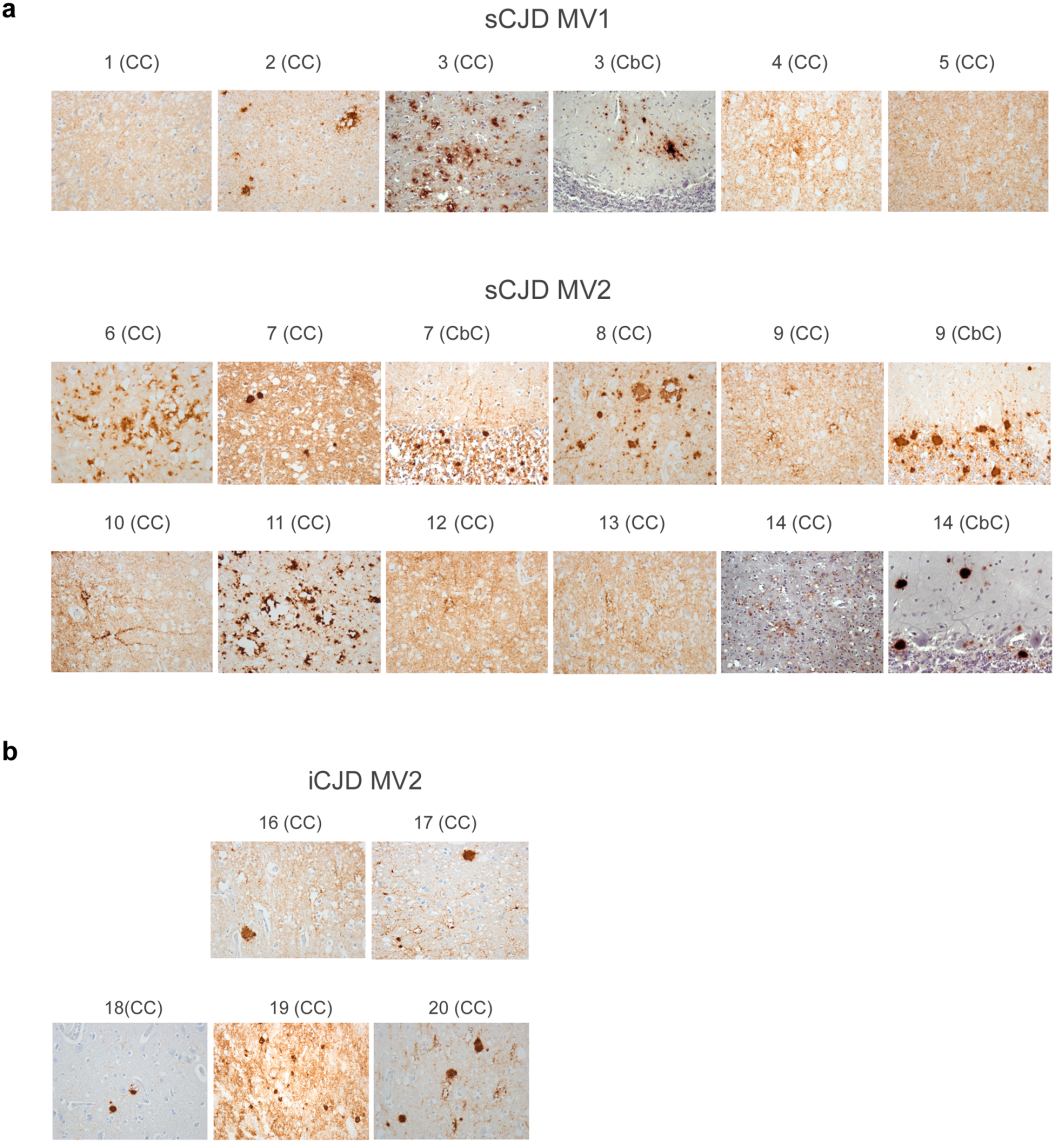
Representative neuropathology of heterozygous (MV) sCJD and iCJD patients analyzed. (a) PrP immunohistochemistry performed on sections of cerebral cortex (CC) or cerebellar cortex (CbC) from sporadic CJD (sCJD) cases 1–5 of the MV1 molecular subtype and cases 6–14 of the MV2 molecular subtype. (b) PrP immunohistochemistry performed on sections of cerebral cortex (CC) from iatrogenic CJD (iCJD) cases 16–20 of the MV2 molecular subtype. Original magnifications were X400. The PrP antibody used was the mouse monoclonal antibody KG9 [34].

**Fig 3:**
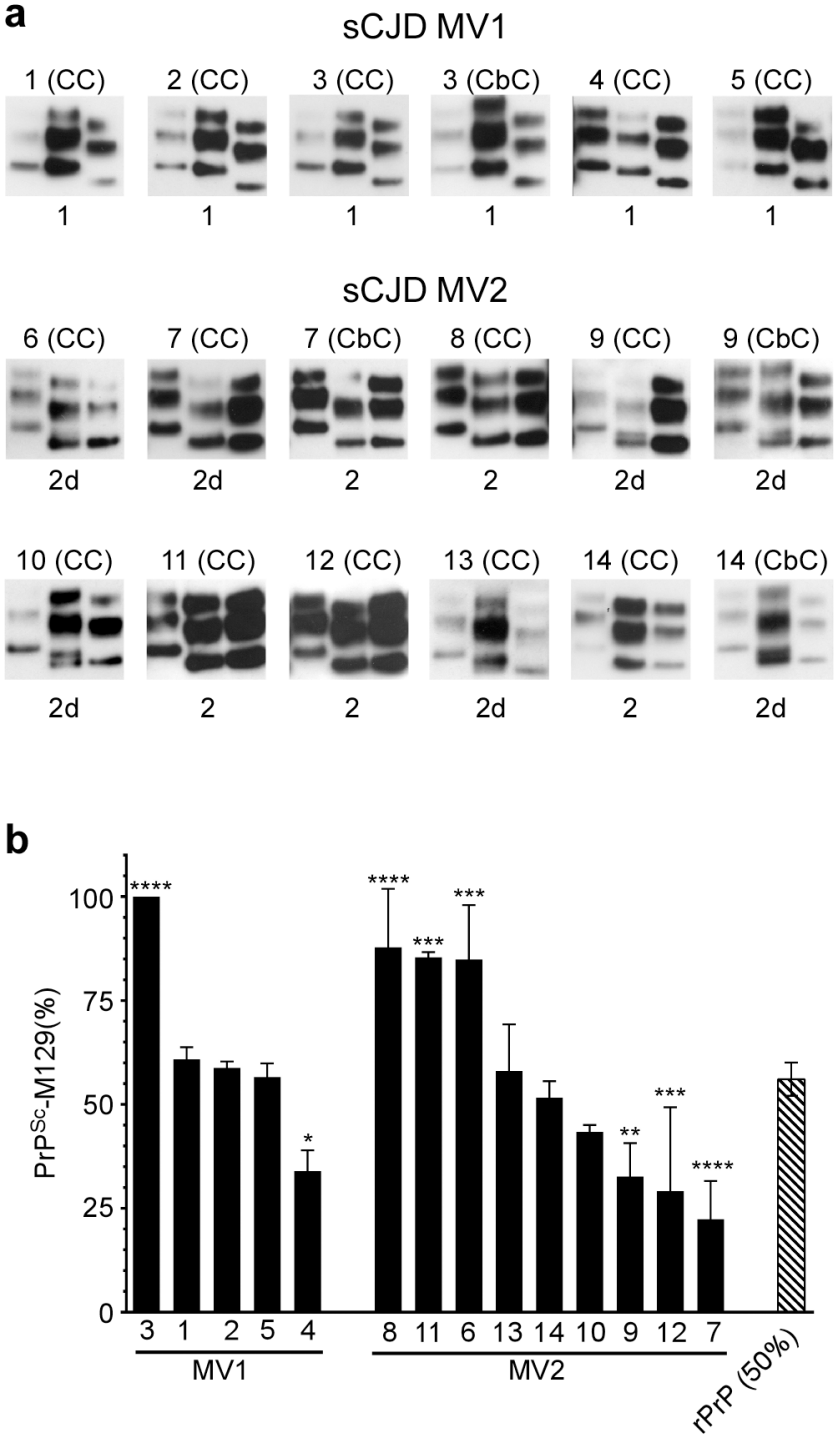
The relative abundance of PrPSc containing M at residue 129 is variable and independent of sCJD type in heterozygous (MV) sCJD patients. (a) Molecular typing of PrP^Sc^ from the brain homogenate of heterozygous sCJD cases 1–14 by the method of Parchi et al. [33]. Each sample (middle lane) is shown flanked by Type 1 (left lane) and Type 2 (right lane) reference standards from sCJD MM1 and VV2 subtype cases, respectively. Protease-resistant PrP from all samples was of the A glycotype in which diglycosylated PrP ≤ monoglycosylated PrP [33]. Case number and brain region are indicated above each blot while PrP^Sc^ type is indicated below. The most informative exposure for the test and reference standard samples from a series of timed exposures is shown. CC = cerebral cortex; CbC = cerebellar cortex. (b) Relative abundance of PrP^Sc^-M129 in MV1 and MV2 sCJD. Data are the percent mean ± SD of PrP^Sc^-M129 (black bars) detected in the CC or CbC (samples 3, 7, 9, and 14 only) of sCJD patients. Each bar represents n = 3–4 technical replicates except for case 4 where n = 2. A stoichiometrically adjusted solution of 50% rHuPrP-M129 and 50% rHuPrP-V129 (rPrP (50%), hatched bar) was used as a control (n = 8). Statistical analysis was done using a 1-way ANOVA with Dunnett’s post-test using rPrP (50%) as the control sample. * p = 0.01 to 0.05, **p = 0.001 to 0.01, ***p = 0.0001 to 0.01, ****p <0.000.

Patient brain samples from 14 cases of heterozygous sCJD were analyzed and their clinical and molecular data are presented in Table 1. The prominent neuropathological features of each case are described in S2 Table and exemplified by images of PrP immunohistochemistry performed on cerebral cortex (CC) and, where relevant to this study, cerebellar cortex (CbC) samples (Fig 2a). No PrP^Sc^ is detected in non-CJD brain using this technique (for an example see S3a Fig). The pathological and molecular features correspond broadly to the histopathological types proposed by Parchi et al [5]. However, in certain cases, such as case 3, there was a discrepancy between molecular and histopathological findings (see S2 Table) suggesting an atypical case of MV1 sCJD. Age of onset for the sCJD cases analyzed ranged from 53–77 years (mean ± SD = 64.7 ± 7.3) while disease duration ranged from 4–21 months (mean ± SD = 10.9 ± 5.7). Molecular analysis had previously identified 5 of the cases as being of the MV1 sCJD subtype (cases 1–5) and 9 as being of the MV2 sCJD subtype (cases 6–14) (Table 1). Western blot analysis comparing a sample from the specimen used for MS (Fig 3a, middle lane of each blot) to reference standards for Type 1 PrP^Sc^ from sCJD MM1 (Fig 3a, left lane of each blot) and Type 2 PrP^Sc^ from sCJD VV2 (Fig 3a, right lane of each blot) confirmed the expected presence of Type 1 or Type 2 protease-resistant PrP in these samples. Analysis of samples from one sCJD MV1 case specimen consistently showed a mobility slightly ahead of the Type 1 reference standard (Fig 3a, case 4). The blots further resolved the presence of a Type 2 doublet (2d) in 6 out of the 9 MV2 subtype cases (Table 1 and Fig 3a). The relative amounts of the two doublets varied between samples from equivalence (case 10 CC) to barely detectable amounts of the least abundant band (case 7 CC). None of the sCJD specimens examined contained both Type 1 and 2 PrP^Sc^ (Fig 3a). No PrP^Sc^ is detectable in PK-treated non-CJD brain using this technique (for an example see S3b Fig).

**Table 1:** Clinical and molecular features of heterozygous (MV) CJD cases analyzed.

Case ID Number	Gender	Age at Onset^a^	Duration^b^	Molecular Sub-classification	Brain Region^c^	PrP^Sc^ Type^d^	M129 (%)^e^
1	M	75	5	sCJD MV1	CC	1	61 ± 3
2	M	77	4	sCJD MV1	CC	1	59 ± 2
3	M	62	14	sCJD MV1	CC	1	98 ± 3
					CbC	1	100 ± 0
4	M	64	14	sCJD MV1	CC	1^f^	34 ± 5
5	F	53	4	sCJD MV1	CC	1	57 ± 3
6	F	61	13	sCJD MV2	CC	2d	85 ± 13
7	F	62	21	sCJD MV2	CC	2d	38 ± 18
					CbC	2	22 ± 9
8	M	75	8	sCJD MV2	CC	2	88 ± 14
9	M	65	21	sCJD MV2	CC	2d	82 ± 8
					CbC	2d	33 ± 8
10	F	60	14	sCJD MV2	CC	2d	43 ± 2
11	M	72	7	sCJD MV2	CC	2	85 ± 1
12	F	64	13	sCJD MV2	CC	2	29 ± 20
13	F	59	18	sCJD MV2	CC	2d	58 ± 11
14	M	57	15	sCJD MV2	CC	2	62 ± 2
					CbC	2d	52 ± 4

^a^Age of onset in years.

^b^Disease duration in months.

^c^CC = cerebral cortex; CbC = cerebellar cortex.

^d^PrP^Sc^ typing according to the method of Parchi et al. [33].

^e^Mean percentage ± SD of PrP^Sc^ with M129. There was no significant correlation of percentage PrP^Sc^-M129 with sCJD type, age of onset, or disease duration (p>0.1).

^f^In one sCJD MV1 case (ID 4) the mobility of the non-glycosylated PrP^Sc^ band was found to be very slightly but consistently greater than that of the Type 1 standard.

PrP^Sc^ enriched from the cerebral cortex or cerebellar cortex of the 14 cases of heterozygous sCJD as well as 3 non-CJD neurological controls was analyzed by MS. For each patient sample, the number of PrP spectra spanning residues PrP111-136 was determined. Using the data analysis criteria described in the materials and methods, an average of >5 PrP111-136 spectra were identified in every sCJD sample examined but not in the non-CJD controls. The abundance of PrP^Sc^-M129 relative to PrP^Sc^-V129 varied between MV1 samples (Fig 3b and Table 1), ranging from 100% to 34% of the total PrP^Sc^ detected. Similar results were obtained for the MV2 samples where the percentage of PrP^Sc^-M129 ranged from 88–22% (Fig 3b and Table 1). For each sCJD molecular type approximately 20–30% of the cases significantly favoured PrP^Sc^-M129, 20–30% favoured PrP^Sc^-V129, and all other cases had an equal distribution of PrP^Sc^-M129 and PrP^Sc^-V129 (Fig 3b).

**Fig 4:**
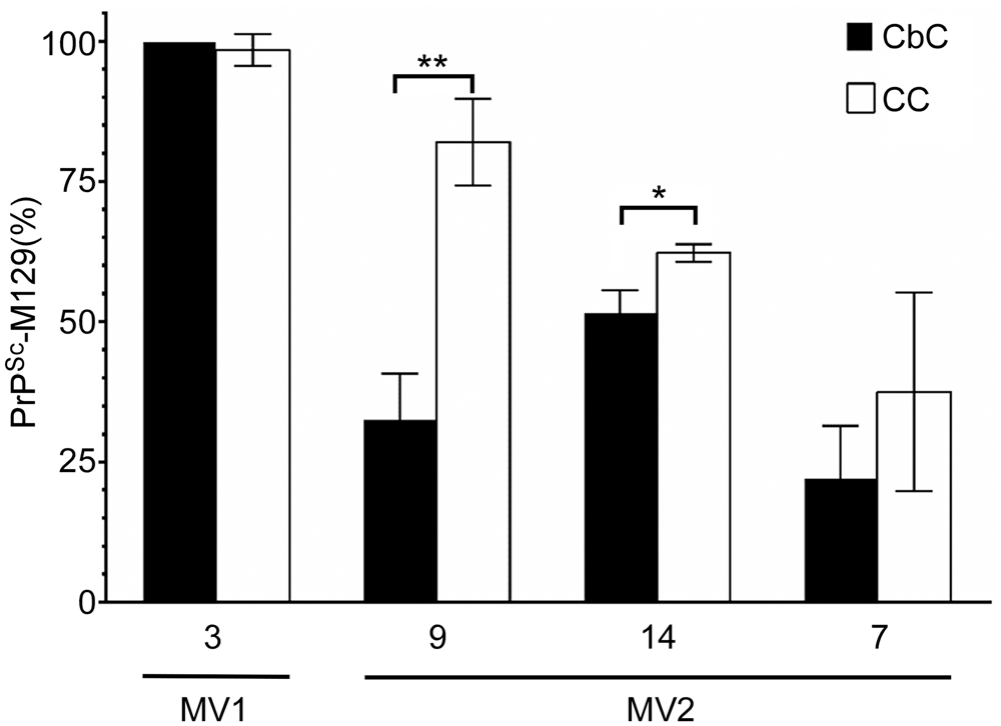
The relative abundance of PrPSc containing M at residue 129 can vary between brain regions in heterozygous (MV) sCJD patients. Data are the percent mean ± SD of PrPSc-M129 detected in the CbC (black bars) or CC (white bars). The case number is indicated under each pair of bars. Each bar represents n = 3–4 technical replicates except for the CC from cases 7 and 9 where n = 2. Statistical significance was determined using the unpaired Student’s t-test. *p = 0.01, **p = 0.002.

We next determined if the relative abundance of PrP^Sc^-M129 and PrP^Sc^-V129 could also vary between brain regions within an individual sCJD patient. The relative abundance of PrP^Sc^-M129 and PrP^Sc^-V129 in the CbC was compared to that of the CC in one MV1 and three MV2 sCJD cases (Table 1). There were no major differences in the amount of PrP^Sc^-M129 (Fig 4) or the type of PrP^Sc^ deposition (Fig 2a) in the cerebral and cerebellar samples from cases 3 and 7. However, the amount of PrP^Sc^-M129 (Fig 4) as well as the type of PrP^Sc^ deposition (Fig 2a) differed significantly between brain regions in the two other cases, 9 and 14. While only 4 sCJD cases were analyzed, the data suggest that within the same patient the amount of PrP^Sc^-M129 and PrP^Sc^-V129 can differ markedly between different regions of the brain with distinctive neuropathologies.

In order to determine if differences in the amount of each allotype were associated with phenotypic variables, the relative abundance of PrP^Sc^-M129 was compared to the sCJD molecular subtype, age of onset, and duration of clinical disease. Statistical analysis revealed no significant correlation between the amount of PrP^Sc^-M129 and sCJD molecular subtype, duration, and age of onset (p > 0.1, Table 1). For example, sCJD MV1 case 3, where 100% of PrP^Sc^ was M129, had a similar age of onset and disease duration to sCJD MV1 case 4 where PrP^Sc^-M129 represented only 34% of the total PrP^Sc^ identified (Table 1). Thus, despite significant differences between cases, the MV ratio of the PrP^Sc^ allotypes did not appear to overtly influence disease onset or progression. There was evidence that PrP^Sc^ allotype might influence disease phenotype in some heterozygous sCJD patients. In two cases, 9 and 14, differences in the PrP^Sc^ allotype ratio between brain regions (Fig 4) were associated with different neuropathologies (Fig 2a). However, when all of the MV sCJD cases were compared, there was no definitive correlation between the amount of PrP^Sc^-M129 and various neuropathological features such as the presence of amyloid plaques, pattern of PrP^Sc^ deposition or type of spongiform change (S2 Table).

### Relative abundance of M and V at residue 129 in PrP^Sc^ isolated from iCJD brain

**Fig 5:**
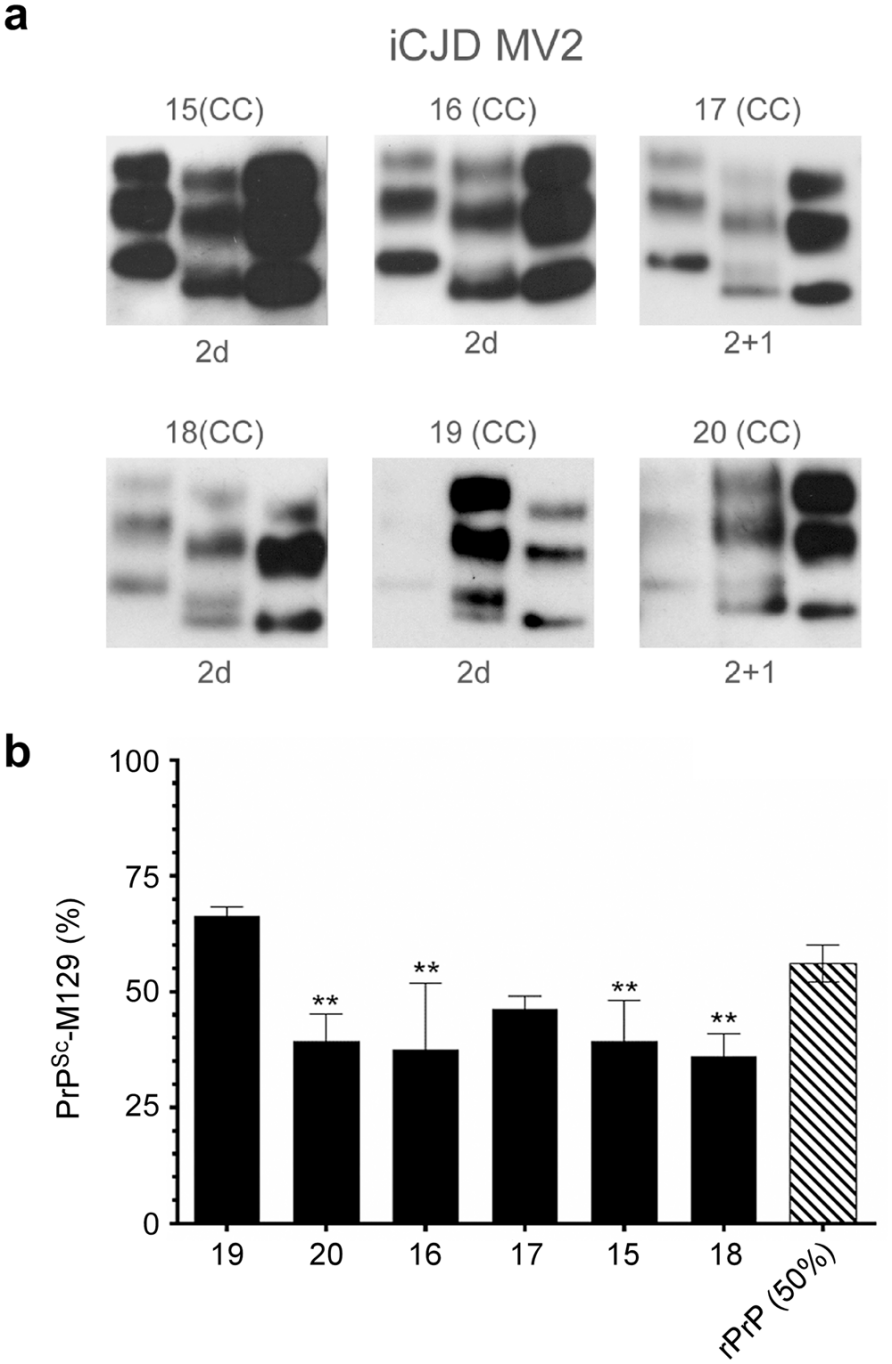
PrPSc containing M at residue 129 is underrepresented in most heterozygous (MV) iCJD patients. (a) Molecular typing of PrP^Sc^ from the brain homogenate of heterozygous iCJD cases 15–20 by the method of Parchi et al. [33]. Each sample (middle lane) is shown flanked by Type 1 (left lane) and Type 2 (right lane) reference standards from sCJD MM1 and VV2 subtype cases, respectively. Case number and brain region are indicated above each blot while PrP^Sc^ type is indicated below. Protease-resistant PrP from all samples was of the A glycotype where diglycosylated PrP ≤ monoglycosylated PrP [33]. The most informative exposure for the test and reference standard samples from a series of timed exposures is shown. (b) Relative abundance of PrP^Sc^-M129 in MV2 iCJD. Data are the percent mean ± SD of PrP^Sc^-M129 (black bars) detected in the CC of iCJD patients. A stoichiometrically adjusted solution of 50% rHuPrP-M129 and 50% rHuPrP-V129 (rPrP (50%), hatched bar) was used as a control (n = 8). Each bar represents n = 3–4 technical replicates. Statistical analysis was done using a 1-way ANOVA with Dunnett’s post-test using rPrP (50%) as the control sample. **p = 0.001 to 0.01.

Next we determined the relative abundance of PrP^Sc^-M129 in brain samples from 6 cases of iCJD, all of whom were heterozygous at codon 129 in *PRNP* (see Table 2 for clinical and molecular data). The prominent neuropathological features of each case are described in S2 Table and exemplified by images of PrP immunohistochemistry performed on cerebral cortex samples in Fig 2b. The mean age of onset for the iCJD cases was younger than that of the sCJD cases, ranging from 27–33 years (mean ± SD of 30.3 ± 2.4) while disease duration was longer than that of the sCJD cases, ranging from 8–32 months (mean ± SD of 17.8 ± 8.5). Western blot analysis of a tissue sample from the specimen used for MS using the method of Parchi et al. [33] showed that Type 2 PrP^Sc^ predominated in all six cases. In 4 of the 6 samples, this took the form of a Type 2 doublet (2d) and in the other two it was found to be a mixture of Type 2 and a smaller amount of Type 1 (2+1) (Table 2 and Fig 5a). As with the heterozygous cases of sCJD, there was no significant correlation between the amount of PrP^Sc^-M129 and disease duration or age of onset (p > 0.3, Table 2). However, as shown in Fig 5b, 4 of the 6 iCJD samples had significantly less PrP^Sc^-M129 than PrP^Sc^-V129. When the average relative abundance of each PrP^Sc^ allotype for all cases was compared between the sCJD and iCJD groups, PrP^Sc^-M129 was significantly less abundant in the iCJD cases than in the sCJD cases (p = 0.01 using the unpaired Student’s t-test). Overall, our results suggest that the composition of PrP^Sc^ among MV heterozygous cases of iCJD is less variable and biased towards a higher proportion of PrP^Sc^-V129 when compared to heterozygous cases of sCJD.

**Table 2:** Clinical and molecular features of heterozygous (MV) iCJD cases analyzed.

Case ID Number	Gender	Age at Onset^a^	Duration^b^	Molecular Sub-classification	Brain Region^c^	PrP^Sc^ Type^d^	M129 (%)^e^
15	M	33	8	iCJD MV2	CC	2d	39 ± 9
16	F	30	12	iCJD MV2	CC	2d	38 ± 14
17	M	32	16	iCJD MV2	CC	2+1	46 ± 3
18	M	28	16	iCJD MV2	CC	2d	36 ± 5
19	F	27	32	iCJD MV2	CC	2d	66 ± 4
20	F	32	23	iCJD MV2	CC	2+1	39 ± 6

^a^Age of onset in years.

^b^Disease duration in months.

^c^CC = cerebral cortex.

^d^PrP^Sc^ typing according to the method of Parchi et al. [33].

^e^Mean percentage ± SD of PrP^Sc^ with M129.

There was no significant correlation of percentage PrP^Sc^-M129 with iCJD type, age of onset, or disease duration (p>0.3).

### PrP^Sc^ allotype ratio and CJD neuropathological subtype

**Fig 6:**
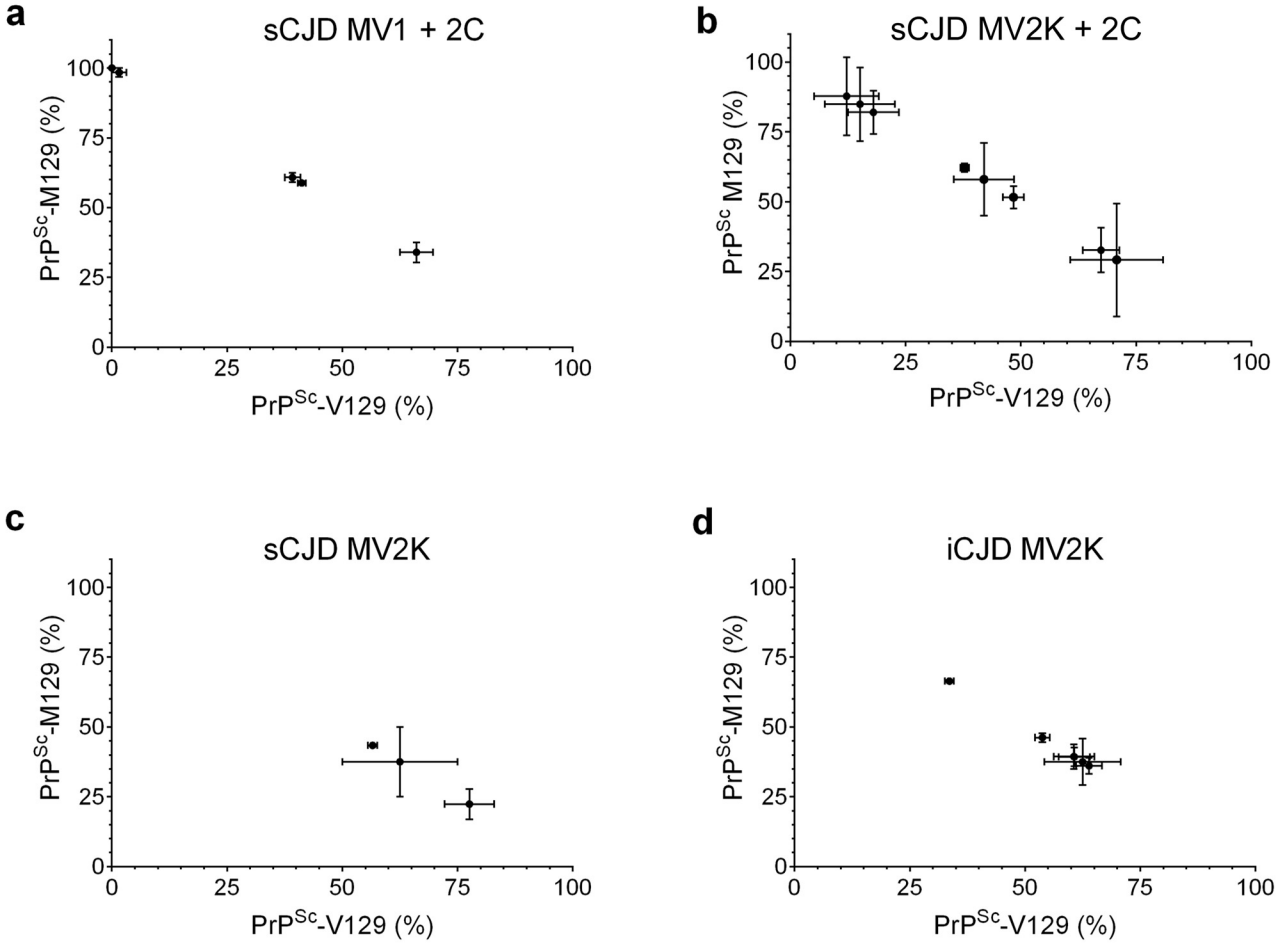
Variable PrP^Sc^ allotype ratios in sCJD neuropathological subtypes. The percentage of PrP^Sc^-M129 versus PrP^Sc^-V129 is shown for neuropathological subtypes for which at least 2 cases were available for comparison. Each point represents the results from a single brain region from a single patient. For some patients, both CC and CbC were available for analysis. (a) sCJD MV1 + 2C, n = 4 cases, 5 samples total. (b) sCJD MV2K + 2C, n = 6 cases, 8 samples total. (c) sCJD MV2K, n = 2 cases, 3 samples total, (d) iCJD MV2K n = 6 cases. Using the unpaired Student’s t-test, the iCJD MV2K PrP^Sc^ allotype ratio differs significantly from both sCJD MV1 + 2C (p = 0.002) or sCJD MV2K + 2C (p = 0.02) but not from sCJD MV2K (p = 0.07).

Epidemiological, genetic and neuropathological data all suggest that human growth hormone associated cases of iCJD in the UK may be the result of contamination with the MV2 or VV2 subtype of sCJD [19, 20]. We therefore compared the PrP^Sc^ allotype ratio to the major neuropathological phenotypes represented in both the sCJD and iCJD cases. As with disease onset and duration, there was no correlation between sCJD PrP^Sc^ allotype and Type 1 and Type 2 sCJD cases with similar neuropathological phenotypes (Fig 6). Both the sCJD MV1 + 2C and MV2K + 2C cases segregated into three distinct groups based on PrP^Sc^ allotype ratio with no significant difference in the mean percentage of PrP^Sc^-M129 between the two types (Fig 6a and 6b). Interestingly, sCJD MV2K and iCJD MV2K had a somewhat similar PrP^Sc^ allotype distribution with no statistically significant difference in the mean percentage of PrP^Sc^-M129 (Fig 6c and 6d). With the caveat that only 2 cases of sCJD MV2K were available for analysis, these data are nonetheless consistent with the hypothesis that UK cases of human growth hormone associated iCJD are the result of exposure to the V2 strain of human prion.

## Discussion

Efficient conversion of PrP^C^ to PrP^Sc^ can be strongly dependent upon homology at a single amino acid residue in PrP^C^ [35], including residue 129 [36, 37], and differences in key amino acid residues can effect disease onset and progression [27, 38, 39]. However, we found no evidence that M or V at residue 129 in PrP^Sc^ correlated with disease onset or progression. This is consistent with some studies of familial human prion disease which also suggest that disease phenotype does not necessarily correlate with the presence of M or V at residue 129 in mutant PrP^C^ [24, 40]. It is possible that, in heterozygous cases of sCJD, the polymorphism at residue 129 does not necessarily lead to protease-resistant PrP^Sc^ conformations that are significantly different. Since PrP^Sc^ conformation is thought to encode prion strain phenotypes [1], PrP^Sc^-M129 and PrP^Sc^-V129 might therefore contribute minimally to conformational differences affecting disease phenotype. Unknown host factors or other forms of abnormal PrP which may or may not be equivalently infectious, such as those with atypical protease-digestion profiles [23] or increased protease-sensitivity [21], may have a more significant influence on age of onset or duration of clinical disease than the presence of an M or V at residue 129 in Type 1 or Type 2 PrP^Sc^.

The epidemiological associations of Type 1 PrP^Sc^ with methionine homozygosity and Type 2 PrP^Sc^ with valine homozygosity within sCJD patient cohorts could be used to argue that the presence of methionine at position 129 of the prion protein predisposes it to misfold to a Type 1 conformation, whereas the presence of valine at the same position predisposes it to misfold to a Type 2 conformation. If this were so, then one might predict that Type 1 PrP^Sc^ in heterozygous sCJD cases would be largely composed of PrP^Sc^-M129 whereas Type 2 PrP^Sc^ in heterozygous sCJD cases would be largely composed of PrP^Sc^-V129 [37]. Data from transmission studies in transgenic mice [11, 12, 41] and in vitro assays of PrP^Sc^ formation [37] are consistent with this prediction. It was therefore surprising that in our study we saw no correlation between PrP^Sc^ type and PrP^Sc^ allotype (Fig 3b). Out of the 5 MV1 sCJD cases analyzed, PrP^Sc^-M129 was the dominant allotype in only one case while PrP^Sc^-V129 was the most prevalent allotype in 3 of the 9 MV2 cases analyzed (Fig 3b). It is therefore unlikely that the amino acid residue at codon 129 in PrP^C^ predisposes to accumulation of either the Type 1 or Type 2 PrP^Sc^ conformations associated with the MM1/MV1 and VV2/MV2 strains of sCJD.

Despite the distinctive transmission properties of MV1 and MV2 sCJD when inoculated into transgenic mice expressing human PrP^C^ [11, 12, 41], the sCJD neuropathological phenotypes MV1 + 2C and MV2K + 2C could not be distinguished based upon their PrP^Sc^ allotype ratios (Fig 6a and 6b). These data are based primarily on the relative abundance of PrP^Sc^-M129 and PrP^Sc^-V129 in the cerebral cortex. It is possible, however, that there may be some correlation with phenotypic differences between brain regions in the same patient. In cases 9 and 14, where distinctive pathologies were observed in the cerebral cortex and cerebellar cortex (Fig 2), the PrP^Sc^ allotype ratios were also significantly different (Fig 4). By contrast, in two cases where neuropathology was similar between these two brain regions (cases 3 and 7, Figs 2 and 4), the PrP^Sc^ allotype ratios were also similar. While these data are suggestive that PrP^Sc^ allotype may correlate with regional differences in neuropathology, further analysis of multiple brain regions from a greater number of patients will be necessary to prove this correlation.

Different strains of sCJD could account for the variability in PrP^Sc^ allotype ratio between patients, although the consistent transmission properties of MV1 and MV2 sCJD into transgenic mice expressing human PrP^C^ argues against this [11, 12, 41]. An alternative explanation for the heterogeneity in the MV PrP^Sc^ allotype ratio may lie in its proposed aetiology. In sCJD the initiating event is thought to be the spontaneous, but stochastic, misfolding of PrP^C^ into PrP^Sc^. Since either PrP^C^-M129 or PrP^C^-V129 could potentially misfold leading to PrP^Sc^ accumulation and disease, the PrP^Sc^ allotype might be expected to vary from patient to patient. Such variation is what we observed in our analysis where the relative abundance of PrP^Sc^-M129 and PrP^Sc^-V129 was independent of sCJD type, differing not only between sCJD cases, but also between brain regions from a single patient. Thus, our data are consistent with either PrP^C^-M129 and/or PrP^C^-V129 refolding into PrP^Sc^ by chance.

Our data may also provide some insight into the relative tendency of PrP^C^-M129 and PrP^C^-V129 to spontaneously adopt the PrP^Sc^ conformation. In approximately one-third of all of the heterozygous sCJD cases analysed, PrP^Sc^-M129 was the most abundant allotype, PrP^Sc^-V129 was the most abundant allotype, or both allotypes were equally represented (Fig 3b). The relatively equal representation of the different possible PrP^Sc^ allotypes does not appear to be consistent with one PrP^C^ molecule being more predisposed to misfold into PrP^Sc^ than the other, as has been suggested by several in vitro studies [42–44]. Rather, our data are consistent with what would be expected if PrP^C^-M129 and PrP^C^-V129 were equally likely to spontaneously misfold into PrP^Sc^. In this context, the differences in relative abundance between the two allotypes could be interpreted as indicating which PrP^C^ molecule initially misfolded into PrP^Sc^. Thus, in cases of heterozygous sCJD where PrP^Sc^-M129 predominates, PrP^C^-M129 could have misfolded into PrP^Sc^ prior to PrP^C^-V129.

However, it is important to note that we have only analyzed a small portion of the brain from the end stage of a long and complex disease process. It is more likely that there are multiple variables, both host and prion specific, that contribute to the relative abundance of each PrP^Sc^ allotype at the end stage of disease. For example, the presence of M or V at residue 129 in PrP^C^ could affect a non-rate-limiting step in PrP^Sc^ formation and accumulation which occurs after the initial misfolding of PrP^C^. A similar hypothesis was proposed by Hosszu et al. who reported that methionine or valine at residue 129 had no measurable effect upon the folding, dynamics, or stability of PrP^C^ [45]. By contrast, there is evidence that there are differences in the stability of the downstream products of PrP^C^ misfolding. PrP^Sc^ from homozygous cases of sCJD demonstrates a broad spectrum of stabilities [21] while short peptide ‘steric zipper’ structures derived from homozygous recombinant PrP molecules form more stable crystalline structures than the hypothetical structures modeled from heterozygous molecules [46]. Therefore, while similar free energy barriers for both PrP^C^-M129 and PrP^C^-V129 would suggest that they may be equally likely to misfold into PrP^Sc^, variability in the thermodynamic stability of the PrP^Sc^ end products may be more important in determining the final relative abundance of each PrP^Sc^ allotype.

In contrast with sCJD, the heterozygous cases of iCJD analyzed were more homogeneous, with a higher proportion of PrP^Sc^-V129 in most of the cases (Fig 5b). As with the sCJD cases, aetiology may provide an explanation for the greater PrP^Sc^ allotype homogeneity of the iCJD samples. In cases of iCJD prior to 2003 which were linked to human growth hormone therapy in the UK, 96% had the VV or MV *PRNP* genotype at codon 129 [19]. In patients diagnosed after 2008, this percentage had decreased to 86% [20]. Based on the earlier cases, Brandel et al. [19] first proposed that the UK cases were caused by either sCJD VV2 or sCJD MV2, a conclusion supported by the more recent study [20]. Our data showing that 1) PrP^Sc^-V129 is the dominant PrP^Sc^ allotype in the majority of the UK human growth hormone-associated iCJD cases analyzed and 2) that the PrP^Sc^ allotype distribution between sCJD MV2K and these cases (Fig 6c and 6d) is very similar, are also entirely consistent with this conclusion.

It is thus possible that the PrP^Sc^-M129 to PrP^Sc^-V129 ratio in heterozygous iCJD patient cohorts might be a means of determining the prion strain that initiated infection. Unlike sCJD, iCJD is a secondary infection of CJD in humans. Selective pressures in the original host with sCJD as well as in the secondary host with iCJD may influence the final PrP^Sc^ allotype ratio. If PrP^Sc^-V129 in the infectious material was of the VV2 type, when transmitted to a second host it would likely interact most efficiently with PrP^C^-V129 to form PrP^Sc^. As a result, PrP^Sc^-V129 would more than likely be the predominant PrP^Sc^ allotype in the infected iCJD patient. Used in this way, mass spectrometry analysis of heterozygous PrP^Sc^ could be the molecular equivalent of in vivo traceback studies that have used the transmission properties of iCJD into transgenic mice [47, 48] or non-human primates [10] to determine the prion strain originally responsible for infection in groups of patients.

## Materials and Methods

### Reagents and supplies

Trypsin was purchased from Promega. Burdick & Jackson water and acetonitrile (ACN) were purchased from VWR. Imperial Coomassie blue stain and iodoacetamide were purchased from Thermo-Fisher Scientific. Formic acid (FA), dithiothreitol (DTT), trifluoroethanol (TFE), phosphotungstic acid (PTA), benzonase, and protein extraction reagent (7M urea, 2M thiourea, 1% C_7_BzO, 40 mM Tris, pH 10.4) were purchased from Sigma-Aldrich.

### Ethics statement

Human brain samples were obtained from the National CJD Research & Surveillance Unit Brain and Tissue Bank in Edinburgh, UK, which is part of Edinburgh Brain Banks. For the purposes of this study, samples were pseudoanonymized using a Brain Bank reference number. Cerebellar and cerebral cortex tissue from two additional sCJD patients were obtained from the University of Verona, Italy. These tissues were obtained at autopsy and sent to the Neuropathology Unit at the University of Verona for statutory definite diagnosis of CJD. All UK cases had consent for research and their supply and use in this study was covered by LREC 2000/4/157 (National Creutzfeldt-Jakob disease tissue bank: acquisition and use of autopsy material for research on human transmissible spongiform encephalopathies, Professor James Ironside, amended date: 9^th^ October 2007). Ethical approval for the acquisition and use of human brain material was obtained from the National Institutes of Health (NIH) Office of Human Subject Research (Exempt #11763 and #12725) and no patient identifiable data was transferred to the NIH.

### Recombinant prion protein (rPrP)

Human recombinant prion proteins spanning residues 23–231 and containing either methionine or valine at residue 129 (rHuPrP-M129 and rHuPrP-V129, respectively) were cloned into a pET41a vector using NdeI-XhoI restriction enzymes. The proteins were expressed as inclusion bodies using *Escherichia coli* Rosetta cells and then purified as described for recombinant hamster PrP [49]. Briefly, guanidine denatured PrP from bacterial inclusion bodies was clarified by centrifugation, bound to NiNTA resin (Qiagen), and then subjected to on-column refolding without reducing agents using a non-denaturing refolding buffer (10mM Tris, 100mM sodium phosphate, pH 8). After elution using refolding buffer adjusted to pH 5.8 and 500mM imidazole, PrP was immediately sterile-filtered, dialyzed into 10mM ammonium formate pH 4.5, and stored at -80°C at a concentration of ~0.3mg/mL until needed. Consistent with our previous results [49], all PrP preparations were approximately 99% pure. In addition, the molecular weight of purified recombinant PrP was verified by intact mass analysis using a Sciex 4000 QTrap system.

### PrP peptides

Synthetic PrP111-136 peptides containing either M or V at residue 129 were diluted in water with 3% ACN/0.1% formic acid and used without further modification after having been subjected to HPLC and MALDI in order to establish purity and the correct molecular weight.

### Allotype ratio concentration curve

Concentrations of purified rHuPrP-M129 and rHuPrP-V129 proteins were determined individually by absorbance at 280nm and then adjusted to contain molar ratios of 0:100, 25:75, 50:50, 75:25, or 100:0 rHuPrP-M129 to rHuPrP-V129, respectively. Final mixtures containing 0.3mg/mL total rPrP were mixed 1:1 with 2X sample buffer (Life Technologies), boiled for 5 min and subjected to SDS-PAGE. Coomassie blue staining displayed a single strong band which was excised and subjected to in-gel trypsin digestion as described below. At least 8 lanes (n = 8–9) were used per individual rPrP mixture and each was subjected to mass spectrometry analysis as described below.

### Human brain tissue

Approximately 2g samples of cerebral cortex (CC) and (in some cases) cerebellar cortex (CbC) from sCJD, iCJD and non-CJD neurological controls were analysed. All cerebral cortex samples were of grey matter enriched frontal cortex with the exception of cases 3 and 14 which were from the occipital cortex. Additionally, grey matter enriched cerebellar cortex samples were analyzes from cases 3, 7, 9 and 14. The choice of frontal cortex reflects both its involvement in MV1 sCJD, MV2 sCJD, and UK human growth hormone-related MV iCJD as well as tissue availability. The selection of cerebellum as a second region to analyze was based on it providing a contrast to the cerebral cortex as well as the known pronounced involvement of the cerebellum in MV2 sCJD.

The diagnosis of definite sCJD or definite iCJD had previously been reached using internationally recognised criteria (http://www.cjd.ed.ac.uk/documents/criteria.pdf) and the cases had been characterised in terms of their neuropathology, *PRNP* codon 129 genotype, and predominant PrP^Sc^ type. The 20 CJD cases examined comprised 14 cases of sCJD in heterozygous patients (5 of the MV1 and 9 of the MV2 molecular subtypes) and 6 cases of iatrogenic CJD in heterozygous patients (all of which were of the MV2 molecular subtype). The three non-CJD neurological cases analyzed were initially suspected of having CJD, but post mortem examination resulted in an alternative diagnosis. These three patients were a 78 year old male with a pathological diagnosis of Lewy body dementia, a 66 year old female with a pathological diagnosis of arteriosclerosis and a seventy year old female with a pathological diagnosis of arteriosclerosis. Western blot analysis had previously failed to detect PrP^Sc^ in the brain in these cases and the *PRNP* codon 129 genotypes of the three patients were VV, MV and MM respectively.

### Neuropathological review

A detailed neuropathological review of the sCJD and iCJD cases analysed in this study was conducted and cases were sub-classified with reference to the sCJD histotypes reported by Parchi et al [5].

### Western blot analysis and typing of PrP^Sc^

To confirm the presence of PrP^Sc^ and to provide a definitive type for the PrP^Sc^ present in the brain specimen being used for mass spectrometry, a 100mg tissue sample was removed from each brain specimen and analysed by western blotting. The stringent sample preparation and proteinase K digestion conditions of Parchi et al 2009 were used [33] that allow distinction to be made between Type 1 (1), Type 2 (2), a mixture of Type 2 and Type 1 (2+1) and doublets of Type 2 (2d).

### Enrichment of PrP^Sc^ using phosphotungstic acid

Brain tissue samples for mass spectrometry were homogenized in phosphate buffered saline (PBS, pH 7.4) to a final concentration of 20% (w/v) using a Mini-BeadBeater-8 (Biospec) and stored in aliquots at -80°C until needed. Enrichment of PrP^Sc^ was performed using PTA precipitation as described previously [50] with some modifications [51]. Briefly, 250 μL of a 20% brain homogenate was diluted to 10% with PBS and then mixed with 500μL of 4% sarkosyl/PBS. Following incubation at 37°C for 30 min, samples were treated with benzonase at 50U/mL for 1 hour and then clarified by a 5 min centrifugation at 5,000 *g*. The supernatants were then digested with PK (50 μg/mL) for 1 hour at 37°C. PTA was added to a final concentration of 0.3% (w/v) and the sample incubated at 37°C for 2 hours. Samples were centrifuged for 30 min at 16,100 x g, the pellets suspended in 200μL of PBS with 200mM EDTA, incubated at 37°C for 30 min, and centrifuged as before. The pellets were resuspended in 200μL PBS, incubated at 37°C for 30 min, and the sample again collected by centrifugation. The final pellet was used for analysis of PrP^Sc^ by mass spectrometry as detailed below.

### Reduction, alkylation and tryptic digestion of PrP^Sc^

Samples of PrP^Sc^ pelleted by PTA precipitation were solubilized in 25μL of protein extraction reagent (7M urea, 2M thiourea, 1% C_7_BzO) and DTT added to a final concentration of 14mM. Following a 30 min incubation at 37°C, iodoacetamide was added to a final concentration of 75mM and the sample incubated in the dark at room temperature for 30 min. The reaction was quenched by the addition of 1M DTT to a final concentration of 200mM followed by the addition of 12μL of 4X NuPAGE sample buffer (Life Technologies). Denatured, reduced and alkylated samples of enriched PrP^Sc^ were briefly heated to 100°C using a heating block prior to loading onto a 10% Bis-Tris 1.5mm gel for SDS-PAGE. Gels were stained with Coomassie blue (Imperial stain, Thermo-Fisher Scientific) but the bands corresponding to PrP^Sc^ were often too faint to be detected by eye. Therefore, the area of the gel lane encompassing molecular mass markers from ~10kDa to 80kDa, including the area of the gel containing PrP^Sc^, was divided into 8 gel slices. In-gel trypsin digestion was then performed as described previously [28]. Each patient sample was analyzed at least 3 times (i.e. 3 technical replicates) as detailed above except for the CC from cases 4, 7 and 9 where only two technical replicates were done. Thus, each technical replicate was composed of 8 individual LC-MS/MS runs corresponding to 8 gel slices excised from a single lane of the Commassie blue stained gel.

### HPLC chip-based nanospray tandem mass spectrometry

Trypsin-digested peptides were identified by LC-MS/MS (abbreviated as MS throughout the manuscript) using an Agilent 1200 HPLC system interfaced with a 6330 XCT Ultra Ion Trap via a chip-cube nanospray source. The mass spectrometer was externally calibrated using a tuning mix provided by Agilent. Data-dependent MS acquisition was performed with dry gas (purified air) set to 4L/min at 350°C, MS capillary voltage 1800V, and a maximum accumulation time of 150ms. The MS scan range was set to 300–1400 m/z in the Ultrascan mode. Four parent ions were selected for each MS/MS cycle with a fragmentation amplitude of 1.0V. Protein digests were loaded onto the HPLC chip (Agilent #G4240-62006, Zorbax 300SB-C18, 5μm, 75μm x 150mm) with an autosampler and washed with Buffer A (3% ACN/H_2_O and 0.1% FA) prior to elution at 300nL/min by reverse-phase chromatography. The gradient was 3–30% Buffer B (90% ACN and 0.1% FA) over 30 min, to 50% B by 40 min, 80% B by 50 min, 90% B by 60 min, 97% B by 70 min, and back to 3% B by 78 min. This run was followed by a 4 min post-run re-equilibration at 3% B with a total run time of approximately 80 min.

### Data analysis

Raw data were processed into MGF peak lists using MASCOT Distiller v2.4.3.0. The MGF files were searched using MASCOT Daemon against a target database (www.uniprot.org) filtered for human taxonomy consisting of 20,235 entries which included an entry for PrP111-136 containing V at residue 129 in addition to the default Swiss-Prot entry containing the full PrP sequence with M at residue 129. The trypsin/P search parameters for MASCOT protein identification consisted of one missed tryptic cleavage allowed with a fixed carbamidomethylation (+57, Cys) and a variable oxidation (+16, Met). Mass tolerances of 2.0 and 1.0 Daltons were used for parent and monoisotopic fragment ions, respectively. The resulting DAT files generated by MASCOT were used as input files for spectral counting using the ProteoIQ bioinformatics platform (Premier Biosoft Inc) [52] with the constraints that only MASCOT ion scores of ≥ 30 and only peptides of ≥ 7 amino acids in length were considered in these calculations. The number of PrP spectra from each sample were then compared to the other samples that had been processed using the same protocols and the same instrument parameters.

In PTA precipitates from the 3 non-CJD brains examined, only 5 PrP peptides were detected in a single sample following a total of 72 MS runs. This indicated that little or no PrP^C^ was present in the final non-CJD PTA pellet. A background threshold of ≥5 total PrP peptides was therefore used to exclude any potential contribution of PrP^C^ to the final PTA pellet and all 3 non-CJD samples were considered negative for PrP^Sc^. By contrast, all PTA precipitated sCJD and iCJD samples had total PrP spectral counts at least 26-fold above the background threshold of 5 spectral counts. Only western blot positive samples yielding greater than 5 PrP111-136 peptides were utilized for semi-quantitation as spectral counts of less than 5 spectra are considered to be unreliable for spectral counting [32, 53].

### Statistics

Mean and standard deviation (SD) were derived using the individual technical replicates described above. The percentage of PrP^Sc^-M129 and PrP^Sc^-V129 was calculated by dividing the number of peptides containing M129 or V129 by the total number of peptides containing residue 129 and multiplying by 100. The statistical significance of multiple data sets was determined using a 1-Way ANOVA with Dunnett’s post-test. A 50:50 mix of rHuPrP-M129 rHuPrP-V129 was set as the control group. The potential relationship between disease onset or duration and the percentage of PrP^Sc^ containing M at residue 129 was analyzed by determining the Pearson correlation coefficient r using correlation analysis. The unpaired Student’s t-test was used to analyze the percentage of PrP^Sc^-M129 between different brain regions and the percentage of PrP^Sc^-M129 in sCJD versus iCJD. All calculations were done using the GraphPad Prism software package, version 6.04.

## Supporting Information

S1 Fig Coverage of human PrP residue 129 by tandem mass spectrometry. Representative MS/MS spectra of the PrP111-136 (+2) peptide from (a) rHuPrP-M129 or (b), rHuPrP-V129, and the synthetic peptide PrP111-136 containing either methionine (c) or valine (d) at amino acid residue 129. The peptide sequence, charge state and observed *m/z* value for the MS1 ion are shown above each mass spectrum. Amino acid residue 129 is underlined. Corresponding *b* and *y* ions with observed *m/z* values matching those calculated *in silico* within the error range of the instrument are labeled. The *m/z* values shown in blue are not affected by residue 129 and therefore are closely matched between the two spectra within the error range of the instrument. By contrast, the *m/z* values shown in red reflect the difference in mass between the methionine and valine residues.
(TIF)

S2 Fig Calibration curve of rHuPrP-M129 in solution with rHuPrP-V129. Recombinant HuPrP molecules containing either M129 or V129 at codon 129 were mixed in the following molar ratios: 0:100, 25:75, 50:50, 75:25 and 100:0 rHuPrP-M129 to rHuPrP-V129, respectively. The mixtures were subjected to SDS-PAGE, stained with Coomassie blue and the excised bands were digested with trypsin. Mass spectra were collected from at least 8 individual LC-MS runs for each formulation. Spectral counts were used to determine the percentage of rHuPrP-M129 in each mixture. Non-linear regression analysis yielded a correlation coefficient of R^2^ = 0.89, indicating a good correlation between the concentration of rHuPrP-M129 in solution and spectral counts. Importantly, there was 100% specificity for the detection of each allotype in solutions containing only M129 or V129 rHuPrP.
(TIF)

S3 Fig Specificity of PrP^Sc^ detection in western blot and immunohistochemistry. Immunohistochemistry (a) and western blot analysis (b) for PrP in frontal cortex specimens from a negative control (non-CJD) case with a pathological diagnosis of Alzheimer’s disease and vascular dementia and a *PRNP* codon 129 MV genotype. Immunohistochemistry for PrP shows haematoxylin counter-stained nuclei (blue) but an absence of PrP staining (brown). Original magnifications X400. The PrP antibody used in immunohistochemistry was the mouse monoclonal antibody KG9 [34]. Western blotting by the method of Parchi et al. [33] shows abundant PrP in the absence of proteinase K digestion (-), but no PrP signal following proteinase K digestion, even when a 10 fold increased amount of brain homogenate is loaded (+). A reference lane of molecular mass markers (M) with their mass indicated in kilodaltons and a lane of proteinase k treated variant CJD brain homogenate (V) are shown for reference. Frozen and fixed tissue from this case were provided by Edinburgh Brain and Tissue Banks (11/ES/0022).
(TIF)

S1 Table Unique PrP peptides commonly identified in a tryptic digest of heterozygous sCJD brain tissue. (DOC)

S2 Table Sub-classification of cases according to neuropathological features. (DOC)
