# Correction: The Distribution of Prion Protein Allotypes Differs Between Sporadic and Iatrogenic Creutzfeldt-Jakob Disease Patients

**DOI:** 10.1371/journal.ppat.1005496

**Published:** 2016-03-08

**Authors:** Roger A. Moore, Mark W. Head, James W. Ironside, Diane L. Ritchie, Gianluigi Zanusso, Young Pyo Choi, Suzette A. Priola

The sixth author’s name is listed incorrectly in the citation. The correct name is Choi YP. The correct citation is: Moore RA, Head MW, Ironside JW, Ritchie DL, Zanusso G, Choi YP, et al. (2016) The Distribution of Prion Protein Allotypes Differs Between Sporadic and Iatrogenic Creutzfeldt-Jakob Disease Patients. PLoS Pathog 12(2): e1005416. doi:10.1371/journal.ppat.1005416
