# Supplementary figures and images for: The Distribution of Prion Protein Allotypes Differs Between Sporadic and Iatrogenic Creutzfeldt-Jakob Disease Patients
Source: PLoS Pathog. 2016 Feb 3;12(2):e1005416. doi: 10.1371/journal.ppat.1005416 (PMC4740439; doi:10.1371/journal.ppat.1005416)

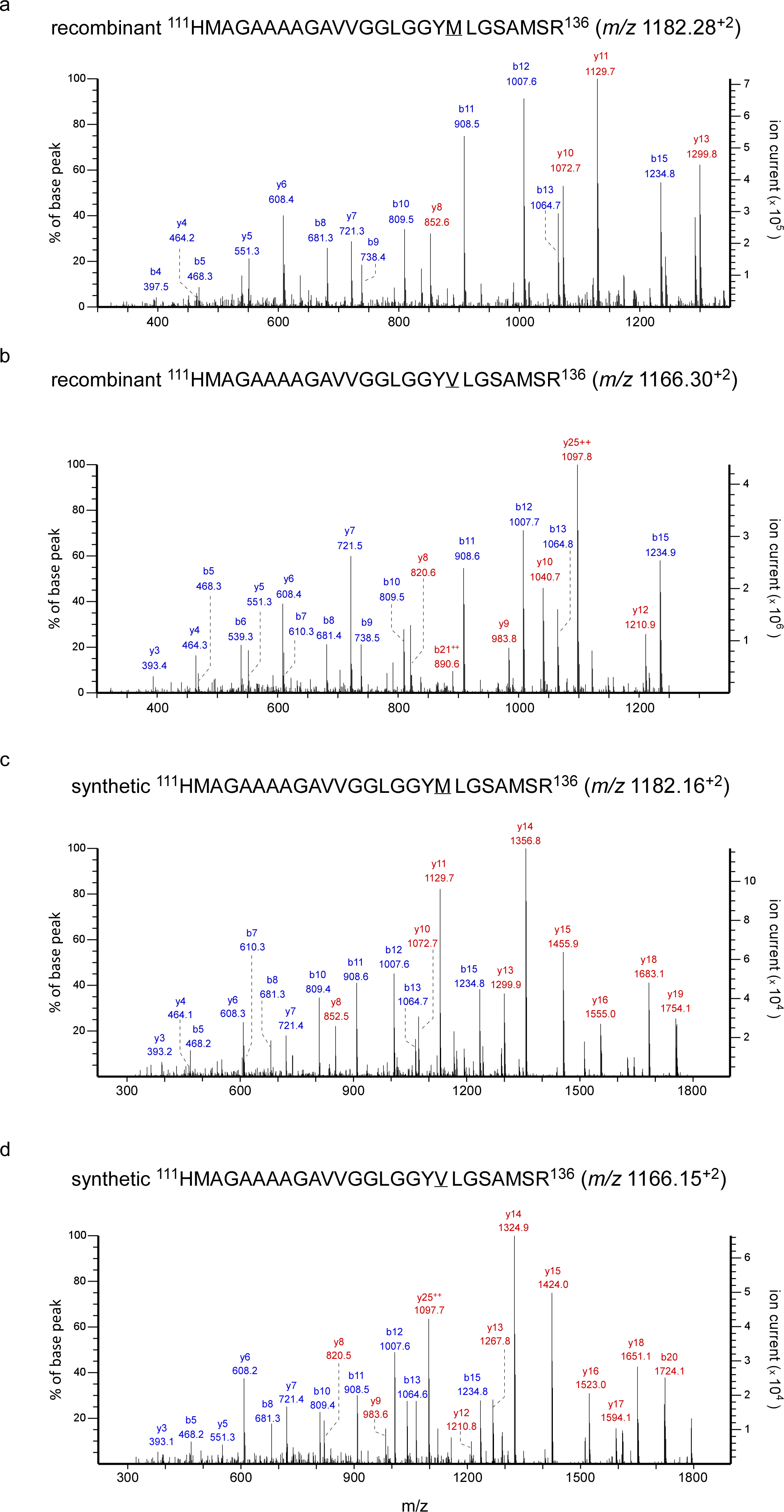

Supplement: S1 Fig — Representative MS/MS spectra of the PrP111-136 (+2) peptide from (a) rHuPrP-M129 or (b), rHuPrP-V129, and the synthetic peptide PrP111-136 containing either methionine (c) or valine (d) at amino acid residue 129. The peptide sequence, charge state and observed m/z value for the MS1 ion are shown above each mass spectrum. Amino acid residue 129 is underlined. Corresponding b and y ions with observed m/z values matching those calculated in silico within the error range of the instrument are labeled. The m/z values shown in blue are not affected by residue 129 and therefore are closely matched between the two spectra within the error range of the instrument. By contrast, the m/z values shown in red reflect the difference in mass between the methionine and valine residues. (TIF) [file ppat.1005416.s001.tif]

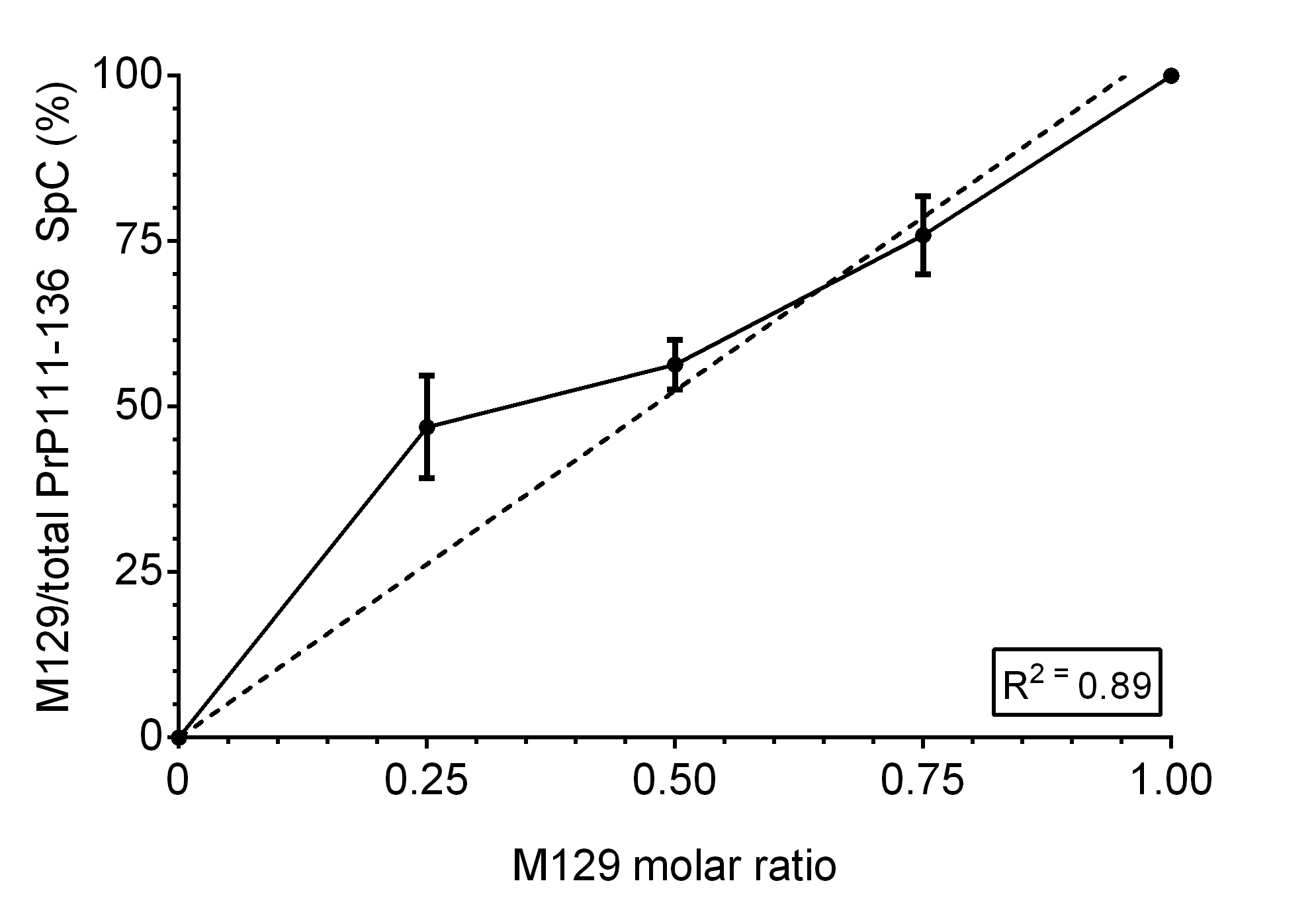

Supplement: S2 Fig — Recombinant HuPrP molecules containing either M129 or V129 at codon 129 were mixed in the following molar ratios: 0:100, 25:75, 50:50, 75:25 and 100:0 rHuPrP-M129 to rHuPrP-V129, respectively. The mixtures were subjected to SDS-PAGE, stained with Coomassie blue and the excised bands were digested with trypsin. Mass spectra were collected from at least 8 individual LC-MS runs for each formulation. Spectral counts were used to determine the percentage of rHuPrP-M129 in each mixture. Non-linear regression analysis yielded a correlation coefficient of R2 = 0.89, indicating a good correlation between the concentration of rHuPrP-M129 in solution and spectral counts. Importantly, there was 100% specificity for the detection of each allotype in solutions containing only M129 or V129 rHuPrP. (TIF) [file ppat.1005416.s002.tif]

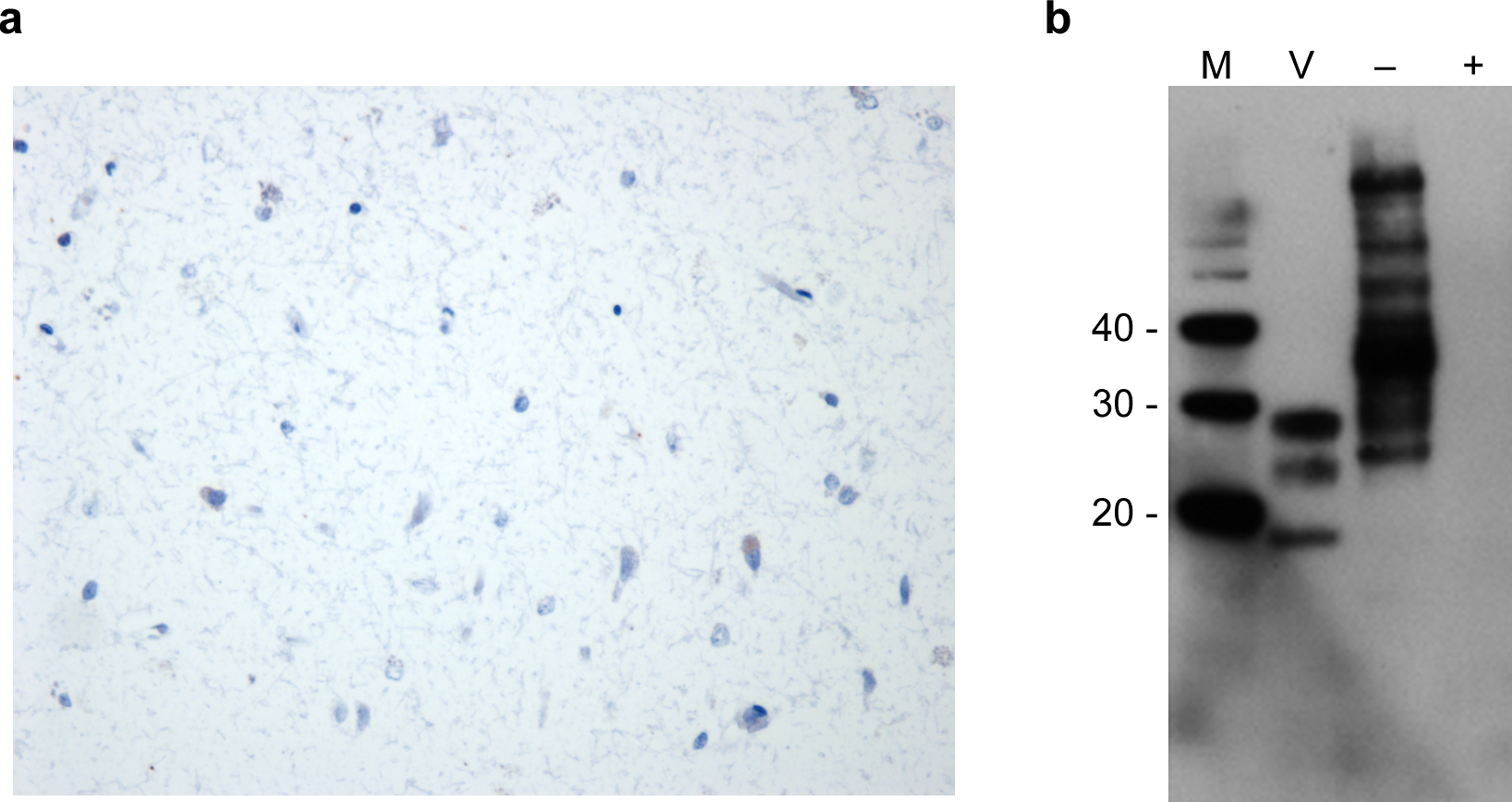

Supplement: S3 Fig — Immunohistochemistry (a) and western blot analysis (b) for PrP in frontal cortex specimens from a negative control (non-CJD) case with a pathological diagnosis of Alzheimer’s disease and vascular dementia and a PRNP codon 129 MV genotype. Immunohistochemistry for PrP shows haematoxylin counter-stained nuclei (blue) but an absence of PrP staining (brown). Original magnifications X400. The PrP antibody used in immunohistochemistry was the mouse monoclonal antibody KG9 [34]. Western blotting by the method of Parchi et al. [33] shows abundant PrP in the absence of proteinase K digestion (-), but no PrP signal following proteinase K digestion, even when a 10 fold increased amount of brain homogenate is loaded (+). A reference lane of molecular mass markers (M) with their mass indicated in kilodaltons and a lane of proteinase k treated variant CJD brain homogenate (V) are shown for reference. Frozen and fixed tissue from this case were provided by Edinburgh Brain and Tissue Banks (11/ES/0022). (TIF) [file ppat.1005416.s003.tif]
