# Supplementary material for: The Distribution of Prion Protein Allotypes Differs Between Sporadic and Iatrogenic Creutzfeldt-Jakob Disease Patients
Source: PLoS Pathog. 2016 Feb 3;12(2):e1005416. doi: 10.1371/journal.ppat.1005416 (PMC4740439; doi:10.1371/journal.ppat.1005416)
Supplement: S1 Table — (DOC) [file ppat.1005416.s004.doc]

**S1 Table. Unique PrP peptides commonly identified in a tryptic digest of heterozygous sCJD brain tissue.**

| **Peptide^a^** | **m/z^b^** | **mw (calc’d)^c^** |
| --- | --- | --- |
| ^111^HMAGAAAAGAVVGGLGGYMLGSAMSR^136^ | 788.382 | 2362.134 |
| ^111^H**M**AGAAAAGAVVGGLGGY**M**LGSA**M**SR^136^ | 804.364 | 2410.119 |
| ^111^H**M**AGAAAAGAVVGGLGGYMLGSA**M**SR^136^ | 799.070 | 2394.124 |
| ^111^H**M**AGAAAAGAVVGGLGGY**M**LGSAMSR^136^ | 799.062 | 2394.124 |
| ^111^HMAGAAAAGAVVGGLGGY**M**LGSA**M**SR^136^ | 799.083 | 2394.124 |
| ^111^HMAGAAAAGAVVGGLGGY**M**LGSAMSR^136^ | 793.656 | 2378.129 |
| ^111^HMAGAAAAGAVVGGLGGYVLGSAMSR^136^ | 777.743 | 2330.162 |
| ^111^H**M**AGAAAAGAVVGGLGGYVLGSA**M**SR^136^ | 788.388 | 2362.152 |
| ^111^H**M**AGAAAAGAVVGGLGGYVLGSAMSR^136^ | 783.075 | 2346.157 |
| ^111^HMAGAAAAGAVVGGLGGYVLGSA**M**SR^136^ | 783.067 | 2346.157 |
| ^137^PIIHFGSDYEDR^148^ | 724.837 | 1447.673 |
| ^157^YPNQVYYR^164^ | 551.732 | 1101.524 |
| ^209^VVEQ**M**CITQYER^220^ | 786.347 | 1570.712 |
| ^209^VVEQMCITQYER^220^ | 778.352 | 1554.717 |
| ^195^GENFTETDVK^204^ | 570.232 | 1138.514 |
| ^a^Peptide numbering and sequence is for human PrP. Methionine sulfoxide residues are shown in bold. Residue 129 is underlined. | | |
| ^b^Experimentally observed mass over charge. | | |
| ^c^The monoisotopic mass calculated from the known peptide sequence. | | |
