# Supplementary material for: The Distribution of Prion Protein Allotypes Differs Between Sporadic and Iatrogenic Creutzfeldt-Jakob Disease Patients
Source: PLoS Pathog. 2016 Feb 3;12(2):e1005416. doi: 10.1371/journal.ppat.1005416 (PMC4740439; doi:10.1371/journal.ppat.1005416)
Supplement: S2 Table — (DOC) [file ppat.1005416.s005.doc]

**S2 Table. Sub-classification of cases according to neuropathological features.**

| **Case ID number**^a^ | | **PrP^Sc^ type in the cerebral cortex by western blot** | **Neuropathological features^b^** | **Sub-classification according to neuropathology^c^** |
| --- | --- | --- | --- | --- |
| 1 | | 1 | Widespread spongiform change in the cerebral cortex of predominantly microvacuolar type and some focal areas of confluent spongiform change. IHC for PrP shows a widespread synaptic/granular distribution with some perivacuolar deposition around areas of confluent spongiform change. No amyloid plaques are visible on routine stains. | MV1 (+2C) |
| 2 | | 1 | Widespread spongiform change in the cerebral cortex of predominantly microvacuolar type and some focal areas of confluent spongiform change. IHC for PrP shows a widespread synaptic distribution with some perivacuolar deposition around areas of confluent spongiform change. No amyloid plaques are visible on routine stains. | MV1 (+2C) |
| 3 | | 1  (1 in cerebellum) | Widespread spongiform change mainly distributed in the deeper layers of cerebral cortex. IHC for PrP shows both granular staining and a perivacuolar pattern. The cerebellum shows mild spongiform change and occasional kuru plaques are visible on routine stains. | Atypical (features of MV1 +2C and MV2K) |
| 4 | | 1^d^ | Widespread spongiform change in the cerebral cortex of predominantly microvacuolar type and some focal areas of confluent spongiform change. IHC for PrP shows a widespread synaptic distribution with some perivacuolar deposition around areas of confluent spongiform change. No amyloid plaques are visible on routine stains. | MV1 (+2C) |
| 5 | | 1 | Widespread spongiform change in the cerebral cortex of predominantly microvacuolar type and some focal areas of confluent spongiform change. IHC for PrP shows a widespread synaptic distribution. No amyloid plaques are visible on routine stains | MV1 |
| 6 | | 2d | Widespread spongiform change in the cerebral cortex with a combination of microvacuolation and some large and often confluent vacuoles. IHC for PrP shows a perivacuolar pattern of deposition with some plaque-like deposits. Severe spongiform change in the cerebellum with kuru plaques visible on H&E stain and following PrP IHC. | MV2K+2C |
| 7 | | 2d  (2 in cerebellum) | Widespread spongiform change in the cerebral cortex and cerebellum. IHC for PrP shows a predominantly synaptic/granular pattern of deposition with some plaque-like deposits. Kuru plaques are a dominant feature in the cerebellum, visible on H&E stain and following IHC. | MV2K |
| 8 | | 2 | Widespread spongiform change throughout the cerebral cortex with a combination of microvacuolation and some large and often confluent vacuoles. IHC shows a composite of granular/synaptic, perineuronal and perivacuolar pattern of PrP deposition. Kuru plaques are a dominant feature in the cerebellum. | MV2K+2C |
| 9 | | 2d  (2d in cerebellum) | Widespread spongiform change throughout the cerebral cortex with a combination of microvacuolation and some large and often confluent vacuoles. IHC shows a composite of granular/synaptic, perineuronal and perivacuolar pattern of PrP deposition. Severe spongiform change is observed in the molecular layer of the cerebellum with the presence of numerous kuru plaques. | MV2K+2C |
| 10 | | 2d | Cerebral cortex shows widespread spongiform change of predominantly microvacuolar type with a combination of granular/synaptic and perineuronal staining of PrP on IHC. The cerebellum shows spongiform change in a patchy distribution with kuru plaques a dominant feature. | MV2K |
| 11 | | 2 | Cerebral cortex shows widespread spongiform change of predominantly confluent type. Strong positive staining for PrP is observed in a predominantly perivacuolar pattern of deposition. The cerebellum is relatively spared. No amyloid plaques are detected. | MV2C |
| 12 | | 2 | Widespread spongiform change throughout the cerebral cortex with a combination of synaptic/granular and perineuronal pattern of PrP deposition. Small focal areas of perivacuolar staining are also observed in the cerebral cortex following PrP IHC. The cerebellum shows some patchy spongiform change in the molecular layer with the presence of numerous kuru plaques. | MV2K (+2C) |
| 13 | | 2d | Widespread spongiform change in the cerebral hemisphere with a predominant perineuronal pattern of PrP deposition on IHC with some focal perivacuolar deposits. Spongiform change is widespread in the cerebellar cortex with the presence of numerous large kuru plaques a dominant feature. | MV2K (+2C) |
| 14 | | 2  (2d in cerebellum) | Widespread spongiform change in the cerebral hemispheres. IHC for PrP shows a perineuronal pattern and some focal perivacuolar deposits. Mild spongiform changes are observed in the cerebellar cortex and numerous and large kuru plaques are a dominant feature. | MV2K (+2C) |
| 15 | 2d | | Widespread and severe spongiform change throughout the cerebral cortex and cerebellum. IHC in the cerebral cortex shows a composite of synaptic and perineuronal PrP deposition with some intensely labelled plaque-like deposits. The cerebellum shows a granular/synaptic distribution with kuru plaques visible on H&E and following IHC. | MV2K |
| 16 | 2d | | Widespread spongiform change throughout the cerebral cortex and cerebellum with a combination of microvacuolation and some large and often confluent vacuoles. IHC shows a composite of granular/synaptic and perineuronal deposition with some intensely labelled plaque-like deposits. Kuru plaques are a predominant feature of the cerebellum. | MV2K |
| 17 | 2+1 | | Widespread spongiform change throughout the cerebral cortex and cerebellum with a combination of microvacuolation and some large and often confluent vacuoles. IHC shows a composite of granular/synaptic and perineuronal deposition. Kuru plaques are observed in the cerebral cortex but are most abundant in the cerebellum. | MV2K |
| 18 | 2d | | Cerebral cortex shows widespread spongiform change of predominantly microvacuolar type with a few plaque-like deposits and some faint perineuronal deposition following IHC. Widespread vacuolation in the cerebellum with numerous kuru plaques visible on H&E and following IHC. | MV2K |
| 19 | 2d | | Status spongiosis in the cerebral cortex with extensive neuronal loss, gliosis and collapse of the cerebral architecture. IHC for PrP shoes a combined synaptic and plaque-like accumulation in the cerebral cortex. Widespread vacuolation in the cerebellum with numerous kuru plaques visible on H&E stain. | MV2K |
| 20 | 2+1 | | Cerebral cortex shows widespread spongiform change of predominantly microvacuolar type with a predominant perineuronal and plaque-like pattern of PrP deposition following IHC. Kuru plaques are a predominant feature of the cerebellum. | MV2K |
| ^a^Cases 1-14 sCJD, cases 15-20 iCJD. | | | | |
| ^b^Abbreviations: IHC=immunohistochemistry, H&E = haematoxylin and eosin. | | | | |
| ^c^Nomenclature in parentheses denote a minor component of the overall neuropathology. | | | | |
| ^d^Slightly increased mobility of non-glycosylated PrP^Sc^ band in this case compared with the type 1 reference standard. | | | | |
